# Epithelioid Hemangioma of Lingual Alveolar Mucosa: An Immunohistochemical Case Report

**DOI:** 10.1155/2014/436240

**Published:** 2014-02-12

**Authors:** Rajat Nangia, Abhiney Puri, Rakhi Gupta, Sucheta Bansal, Amita Negi, Isha Chauhan

**Affiliations:** Department of Oral and Maxillofacial Pathology, Himachal Institute of Dental Sciences, Rampur Ghat Road, Paonta Sahib, District Sirmour, Himachal Pradesh 173025, India

## Abstract

Epithelioid hemangioma is a rare benign vascular lesion that presents as a nodular lesion in the skin of head and neck region. It is a superficial vascular entity which can either be due to tumor or reactive lesion, but the exact etiology is still unknown. We hereby present a rare case which has been reported with the history of small nodular-like growth on mandibular buccal and lingual area. The excisional biopsy was performed and tissue was submitted for histopathological diagnosis. The immunohistochemistry was performed to check the expression of CD31 marker which proved that origin of epithelioid cells was vascular.

## 1. Introduction

Hemangiomas are the benign vasoformative tumors, characterized by an increase in number of blood vessels especially veins and capillaries in a focal area of submucosal connective tissue and are self-involuting in nature. The “hemangioma” in Greek means *haema*: “blood”, *angeio*: “vessel”, and *oma*: “tumor,” that is, a blood vessel tumour. Oral hemangiomas represent 14% of all human hemangioma. There are various subtypes of hemangioma like capillary hemangioma, lobular hemangioma, cellular hemangioma, and epithelioid hemangioma.

Epithelioid vascular tumors are a diverse group of lesions which are characterized by endothelial cells which are epithelioid in appearance. These include three variants: benign vascular tumors or epithelioid hemangioma, borderline tumors, that is, epithelioid hemangioendothelioma, and malignant tumors—epithelioid angiosarcoma [1].

Epithelioid hemangioma (EH) is an uncommon lesion, which occurs with greater frequency in the head and neck region [2]. Wells and Whimster first reported it as angiolymphoid hyperplasia with eosinophilia (ALHE) in 1969 and Rosai et al. termed it as histiocytoid hemangioma in 1979. The term epithelioid hemangioma was first used by Enzinger and Weiss in 1983 to describe the benign counterpart of the vascular lesions [3].

Epithelioid hemangioma typically arises on the head and neck, but the intraoral sites which are more frequently affected are lips, buccal mucosa, and tongue. The extracutaneous sites which are more commonly affected are bone, salivary gland, and muscular area or extremities. There are only limited intraoral cases of EH and only 16 cases have been reported in the English literature [4].

The etiology and pathogenesis of this vascular entity is still uncertain. This entity typically presents as a small angioma-like nodule, red to brown in color and may be located intradermally or subcutaneously in young adults. EH can mimic lymphoproliferative disorders, especially when the lesion arises in atypical location such as the extremities [5].

We hereby report a case of epithelioid hemangioma involving the lingual mucosa in a 30-year-old female patient with local recurrence.

## 2. Case History

A 30-year-old female reported to the outpatient department of oral medicine and radiology for the complaint of a painful swelling in the lower front region since 20–25 days duration. History revealed that the growth increased rapidly in the first few days and there is no alteration in size since then. Patient had difficulty in speaking and eating, due to the growth. The patient was six-month pregnant and the patient did not give any history of trauma to the involved area.

**Figure 1:**
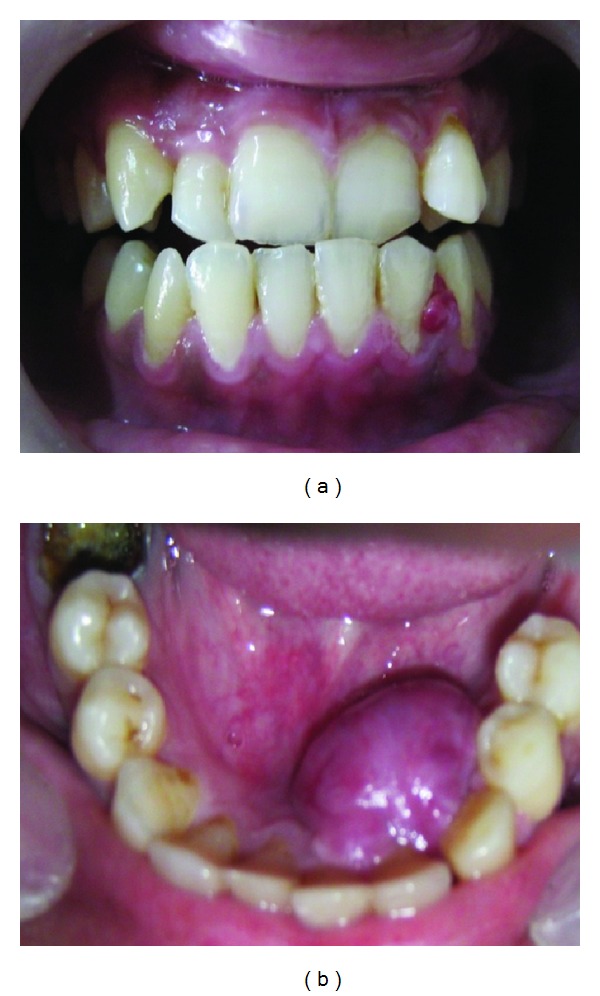
Clinical picture. (a) shows small growth buccally between 32 and 33. (b) shows growth in lingual alveolar mucosa.

Extraoral examination of the head and neck did not show any abnormalities. On intraoral examination soft painless mobile and nodular growth measuring 1 cm × 2 cm was present in the lingual alveolar mucosa of 32, 33, and 34 region. A soft tissue mass measuring 4-5 mm was also present on the labial surface in between 32 and 33 (Figures 1(a) and 1(b)).

There was slight bleeding on brushing and difficulty in speaking. The lesion was erythematous and the tooth adjacent to the mass had no caries and no periodontal pocket. The patient was nondiabetic and nonhypertensive. There was no associated regional lymphadenopathy. Radiographic findings showed no overt destruction of alveolar bone in the area of soft tissue enlargement. Blood investigation revealed raised level of ESR. Blood count did not show eosinophilia (Table 1). The provisional diagnosis of pyogenic granuloma was given.

**Figure 2:**
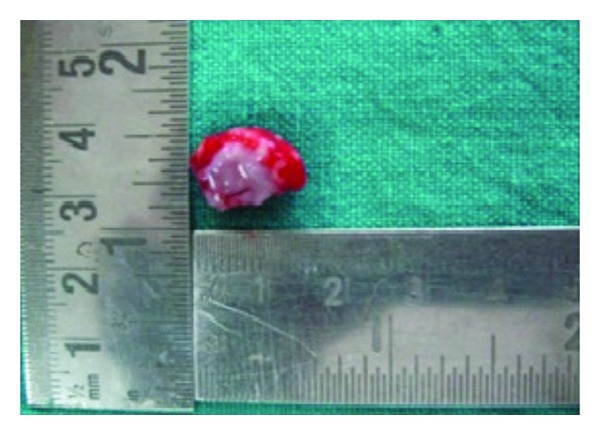
Tissue after excision.

The excision of the lesion was done with proper margins under the lingual nerve block. The two soft tissues of size 0.8 cm × 1.8 cm (Figure 2) and 4 mm × 3 mm, respectively, were submitted to the department of oral pathology and microbiology for microscopic examination.

## 3. Material and Methods

### 3.1. Materials Used

- Automated tissue processor (Yorco).
- Semiautomatic microtome (Leica).
- Hematoxylin and eosin (H&E) staining kit (Nice).
- E-Z antigen retrieval microwave (Biogenics).
- CD31 antibody kit (Dako).

### 3.2. Methods

Routine two-day tissue processing of both the tissues was done using the automated tissue processor; after that the tissues were embedded in paraffin wax; 4-micrometer thick sections of the tissue were cut from the wax block and were placed on two poly-L-lysine coated slides. The routine H&E staining of the slide was done using the H&E staining kit.

The immunohistochemistry of the second slide was done. The sections were deparaffinized in xylol and then they were rehydrated in graded alcohol series. Endogenous block was done using 3% H_2_O_2_ in methanol. The sections were washed in distilled water and antigen retrieval was done in E-Z antigen retrieval microwave as per supplier's instructions (in citrate buffer 10 mM, at pH 6). The slides were then incubated at 20°C for 45 min with monoclonal CD31 antibody. The slides were then incubated with anti-mouse biotinylated bridging antibodies (1/200 dilution) for 30 min. Sections were then washed and incubated with standard avidin-biotin complex (Dako) for 30 min. Antibody binding was seen using H_2_O_2_ as a substrate and diaminobenzidine as chromogen. The slides were then counterstained using hematoxylin.

## 4. Results

**Figure 3:**
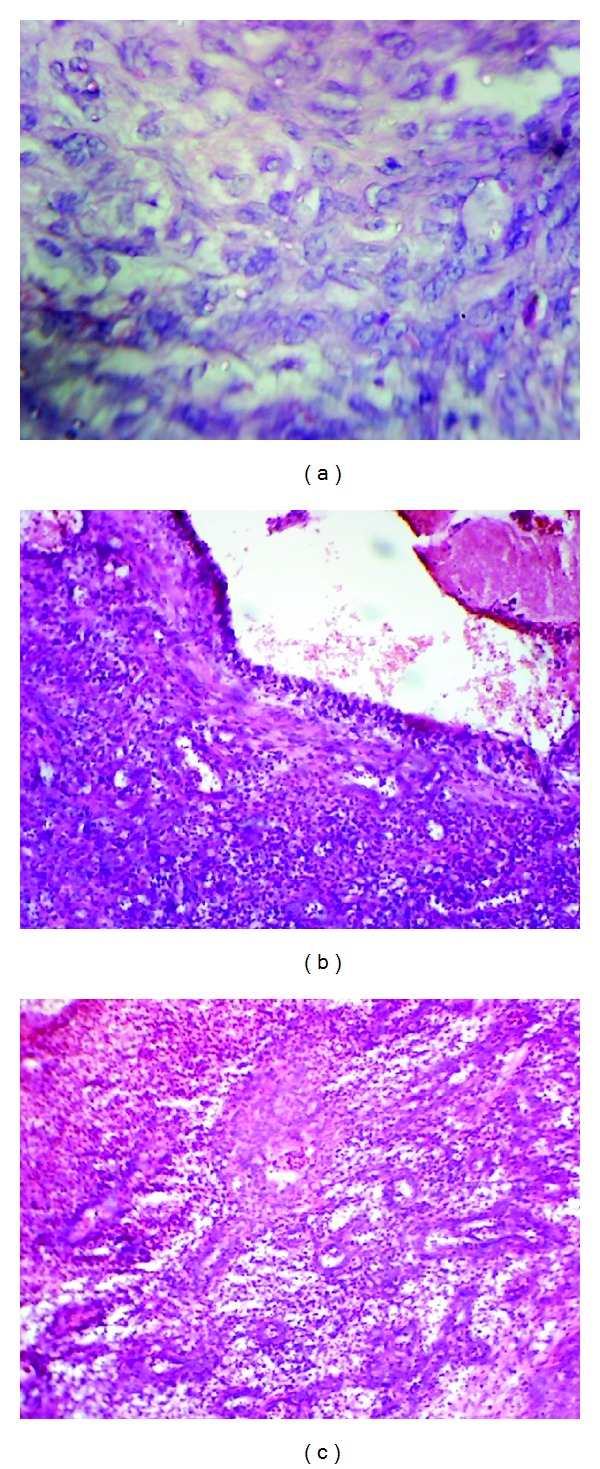
(a) Histopathological picture showing sheets of epitheliod cells (arrows); H&E, ×400 (original magnification). (b) Histopathological picture showing plump epithelioid cells lining blood vessel in tombstone appearance (arrows); H&E, ×100 (original magnification). (c) Histopathological picture showing eosinophils in connective tissue stroma (arrows); H&E, ×400 (original magnification).

Microscopic examination of the biopsy specimen revealed focally ulcerated stratified squamous epithelium overlying the connective tissue stroma. At a few places there were sheets of epithelioid cells. Atypical rounded cells with central nucleus were seen proliferating in the connective tissue mass (Figure 3(a)). Connective tissue stroma was full of loose collagen fibril bundles with underlying proliferation of variably sized blood vessels which were lined by plump endothelial cells giving the “tombstone” appearance (Figure 3(b)). Some markedly dilated spaces contained thrombi. A dense cellular inflammatory infiltrate, mainly lymphocytes, plasma cells, and eosinophils and mast cells were found surrounding the vessels (Figure 3(c)). There were no signs of mitosis and atypical cells.

**Figure 4:**
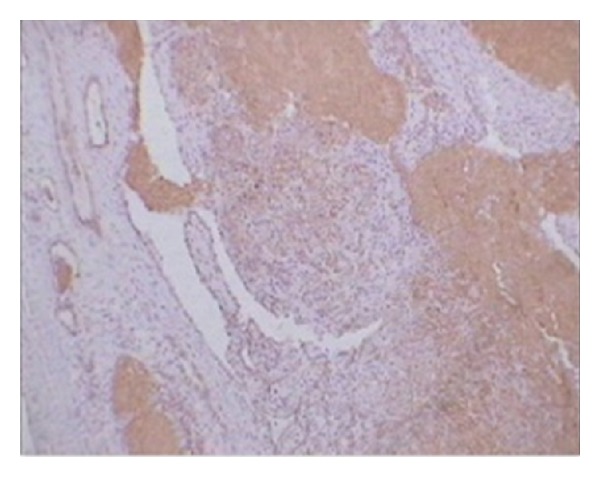
Immunohistochemical picture showing positivity for CD31.

The immunohistochemistry revealed the positivity of these tombstone appearing cells for CD31 proving that the epithelioid cells seen are vascular in origin (Figure 4). A final diagnosis of epithelioid hemangioma was made based on these features.

The patient was kept on regular followup. After a month, a similar lesion reoccurred at the same site. Excision of the swelling was again done with wider margins this time. The tissue was subjected to histopathological examination and it revealed the same features.

## 5. Discussion

Epithelioid hemangioma in the oral mucosa is a rare disease and often confused with other epithelioid vascular tumours and nonvascular soft tissue tumours. In the literature various terms have been used by different authors to describe this lesion, including angioblastic hyperplasia with eosinophilia (ALHE), nodular angioblastic lymphoid hyperplasia with eosinophilia, lymphofolliculosis, histiocytoid hemangioma pseudopyogenic granuloma, and atypical pyogenic granuloma. In 1982, a broad term, epithelioid hemangioma, is given to describe angiolymphoid hyperplasia with eosinophilia and some other vascular diseases [5]. But the diagnosis of epithelioid hemangioma is based upon its histopathological findings. The cases reported so far have been observed to be more common in Asians and Caucasians [6]. It is seen more often in patients of age group 30–33 years.

Oral cases of epithelioid hemangioma are more common in men, mostly at young age (male : female = 23 : 13). The lips are the most frequent site (16 out of 36), followed by the tongue (10 out of 36), buccal mucosa (6 out of 36), and palate (3 out of 36) [6]. Only 21 cases of epithelioid hemangioma have been reported in the oral cavity and the gingiva is the most common site [4]. One case has been reported by Misselevich et al. to affect alveolar mucosa and gingiva by the direct extension from lesion in mucobuccal fold [7].

The extraoral sites of EH are periauricular region, forehead, and scalp. Few cases with multiple lesions have been reported. In 20% of the cases, the lesion is associated with lymphadenopathy and blood eosinophilia [3].

The etiology and pathogenesis of epithelioid hemangioma are unknown. There is a controversy among various authors whether epithelioid hemangioma is a true neoplastic growth or a reactive lesion. According to Martin-Granizo, microtrauma can be a cause of epithelioid hemangioma [8]. It is possible that reactive condition caused by trauma may lead to arteriovenous malformation trigger cellular proliferation and growth secondary to damage or repair of an artery or vein. Epithelioid hemangioma can occur in pregnant women [9]. The cause for this may be attributed to the outbursts of hormones, namely, progesterone, during pregnancy. In our case trauma during the period of pregnancy may have exaggerated the response, leading to the exophytic and nodular growth. Other etiological factors which have been suggested are low grade infections, allergy, trauma, overgrowth of atypical endothelial cells, and inflammatory skin manifestations [3]. Rajendran and Sivapathasundram suggested hypersensitivity response to be the cause of EH on account that there is raised level of eosinophils seen in some of the patients [6].

Peters et al. reviewed all the cases of the literature and found that, histologically, the vessels were lined by epithelioid or histiocytoid endothelial cells which extend into the lumen and impart them a “tombstone” appearance. These epithelioid cells had rounded nuclei and abundant eosinophilic cytoplasm and in some cases there were large vacuoles. The stroma of the tumour was filled with fibromyxoid tissue with a mixed chronic inflammatory cell infiltrate, including eosinophils, lymphocytes, macrophages, and mast cells and occasional germinal centers were seen [10].

Epithelioid hemangioma is differentially diagnosed from other epithelioid vascular tumours and nonvascular soft tissue tumours showing epithelioid characteristics. Macroscopically, EH should be differentiated from Kimuras, disease, salivary gland tumour, squamous cell carcinoma, lymphoma, and pyogenic granuloma and histologically these are differentiated from bacillary angiomatosis, epithelioid angiosarcoma, epithelioid neurofibroma, epithelioid schwannoma, epithelioid fibrosarcoma, Kimura's disease, and epithelioid hemangioendothelioma [3]. There has been a debate regarding the relation between EH and Kimura's disease but the recent studies clearly indicate that the two diseases are different entities. In Kimura's disease, prominent cellular area of lymphocyte forming follicles is seen, which is surrounded by inflammatory infiltrate with blood eosinophilia and fibroma. whereas in epithelioid hemangioma there is proliferation of endothelial cells lining the blood vessels, with abundant eosinophilic cytoplasm [11].

Bacillary angiomatosis is an infectious disease which is caused *Bartonella henselae* or *Bartonella quintana*. Histologically it presents with abundant neutrophils with nuclear dust and clumps of bacteria [12]. Epithelioid angiosarcoma presents as an infiltrative, destructive growth with markedly pleomorphic cells. Cells show atypical mitosis and necrosis [13]. Epithelioid hemangioendothelioma is composed of short cord of spindle shaped endothelial cells. In this lesion well-defined vascular channels are not seen. Endothelial cells show mild pleomorphism and hyalinzed or myxoid stroma [14].

Immunohistochemistry is a technique for identifying cellular or tissue constituents (antigens) by means of antigen-antibody interaction, the site of antibody binding being identified either by direct labeling of the antibody or by use of a secondary labeling method [15]. The immunohistochemistry of epithelioid hemangioma with a vascular marker will show that these epithelioid cells are vascular in origin. CD31 is a vascular marker which was used in our study and result was positive for these epithelioid cells.

As the patient was pregnant it was thought that it can be pyogenic granuloma; moreover, its recurrence after excision also supported it but the histological picture and immunohistochemistry results contradicted the provisional diagnosis.

The treatment should be started once the definitive diagnosis has been made. The treatment for epithelioid hemangioma is complete surgical excision of the lesion with conservative margins and regular followup. Other treatment options which have been successfully used for cases of epithelioid hemangioma are laser cauterization, diathermy, cryotherapy, pentoxifylline, and intralesional corticosteroids and in some cases success has been seen with oestrogen therapy [14].

Recurrence has also been observed in about 20% of the reported cases of epithelioid hemangioma of oral cavity [6].

## 6. Conclusion

By studying this case we conclude that epithelioid hemangioma although a rare entity with only a few cases of recurrence was presented in our patient supported by clinical and histopathological findings. Moreover, epithelioid hemangioma should be taken into consideration while differentiating any peripheral intraoral lesion in pregnant patients.

## Figures and Tables

**Table 1:** Blood investigations.

Hemoglobin—11.2 gm%	
Total leukocytic count—8950/mm^3^	
Differential leukocytic count	
Neutrophils—68%	
Lymphocytes—29%	
Monocytes—01%	
Eosinophils—02%	
Bleeding time—1′30′′	
Clotting time—5′30′′	
